# Remote harm reduction services are key solutions to reduce the impact of COVID-19-like crises on people who use drugs: evidence from two independent structures in France and in the USA

**DOI:** 10.1186/s12954-023-00732-x

**Published:** 2023-01-07

**Authors:** Magally Torres-Leguizamon, Jamie Favaro, Dan Coello, Emmanuel G. Reynaud, Thomas Néfau, Catherine Duplessy

**Affiliations:** 1 SAFE, 11 Avenue de la Porte de la Plaine, 75015 Paris, France; 2 NEXT Harm Reduction, New York, USA; 3 School of Biomolecular and Biomedical Science, University College Dublin, Dublin, Ireland

**Keywords:** COVID-19 pandemic, HaRePo, NEXT Distro, Lockdown, Remote service, Harm reduction, Needle and syringe program

## Abstract

**Background:**

Harm Reduction (HR) policies for People Who Use Drugs (PWUD) have a significant positive impact on their health. Such approaches limit the spread of infections and reduce opioid overdose mortality. These policies have led to the opening of specialized structures located mainly in big cities and urbanized zones. The COVID-19 pandemic reduced access to HR structures in locations undergoing lockdown. Before the pandemic, HR services in France and in the USA were complemented by the development of remote HR programs: HaRePo (Harm Reduction by Post) for France, implemented in 2011, and NEXT Distro for the USA founded in 2017. These programs are free and specifically designed for people who have difficulties accessing HR tools and counseling in-person. PWUD can access HaRePo program by phone and/or email. NEXT Distro users can access the program through its dedicated website. The aim of the study is to test if and possibly how COVID-19 pandemic and the associated lockdowns have impacted the HR services in both countries.

**Methods:**

By using *t*-test comparing the year 2019 with the year 2020, we analyzed how lockdowns impacted the number of new users entering the programs, as well as the numbers of parcels sent and naloxone distributed, by using records of both structures.

**Results:**

We showed that the activity of both programs was significantly impacted by the pandemic. Both show an increase in the number of new users joining the programs (+ 77.6% for HaRePo and + 247.7% for NEXT Distro) as well as for the number of parcels sent per month (+ 42.7% for HaRePo and + 211.3% for NEXT Distro). It shows that remote HR was able to partially compensate for the reduced HR activities due to COVID-19. We also observed that the distribution of naloxone per parcel tends to increase for both structures.

**Conclusion:**

With the ability to reach PWUD remotely, HaRePo and NEXT Distro were particularly effective at maintaining service continuity and scaling up services to meet the needs of PWUD during the COVID-19 pandemic. By studying two independent structures in France and in the USA sharing similar objectives (remote HR), we showed that this approach can be a key solution to crises that impact classical HR structures despite various differences in operating procedures between countries.

## Background

The coronavirus disease (COVID-19) pandemic that started in 2019 had consequences on the daily life of everyone including PWUD through lockdowns, social distancing, impact on economy and by generating stress [1]. These populations had to face both consequences of their addiction and consequences of COVID-19, making them a particularly vulnerable population. For instance, in England fewer HR materials were distributed with potential increase of material reuse and sharing [2]. In other countries, closing HR structures induced higher overdoses during the pandemic [3]. Nevertheless, COVID-19’s impact on HR services depended on the regulating rules implemented by the states or countries. For instance, in Sweden where the COVID-19-related policies were less restrictive compared to other countries like France, Spain, Germany, Belgium, USA, etc., the impact on HR services were relatively limited [4], whereas in other countries like Iran, Portugal or Spain the restrictions impacted much more drastically the HR structures [5–8].

Due to the COVID-19 related restrictions, several HR services had to adapt their activities to be able to keep delivering paraphernalia with social distancing, telehealth or restricted access opening hours [9–11]. Some HR structures developed creative solutions. For instance, in Los Angeles, an HR program implemented supply distribution through telephone booths to support homeless PWUD [12]. Within the new alternatives proposed to adapt HR services to the COVID-19, telehealth services have been one of the most developed [13]. In several countries, telehealth limited the impact of COVID-19-related restrictions on PWUD but only partially solves their difficulties. In particular, those who need paraphernalia distribution are still affected by the pandemic restrictions with less material available. In this context remote HR seems to be a promising avenue [14] combining the benefits of telehealth but with the logistic structure and funding to send the paraphernalia by mail. These remote HR structures were initially developed to access populations difficult to reach. Despite the many HR programs that exist in the world, there are still a significant number of drug users who do not have access to this type of service [1]. The difficulties of access are varied and sometimes multiple. For example, they may live in rural areas and/or have difficulties in traveling to the nearest HR structure, or have working hours that are incompatible with the opening hours of the structures. Sometimes, HR structures or Syringe Service Programs (SSPs) do not offer the amount and/or variety of tools needed by users. Consumers may also be afraid of being judged and/or the lack of confidentiality (e.g., in pharmacies or small towns). Finally, the cost of equipment in pharmacies and lack of knowledge about the HR system can also be a barrier to accessing equipment.

It is important to note that COVID-19 pandemic consequences on HR services (e.g., structures closed, difficulties to attend in person) were observed before (example of the Hurricane Sandy in NY in 2012) and might be occurring again in the future [15, 16]. Indeed, big events, in particular related to global changes, are expected in the coming decades [16]. Thus, the COVID-19 crisis can also be analyzed using the concept of big events defined as ﻿any social crises due to diseases, climate events, terrorist attacks, and other phenomena that negatively affect general and individual behaviors, with possible consequences on PWUD health through an impact on HR structures [17, 18].

In this context, we assumed that remote HR service can be a key resource to mitigate the big events impact on PWUD whatever the local context and we compared how two remote HR services in France and in the USA (HaRePo and NEXT Distro [19, 20]) were impacted by the COVID-19 pandemia.

**Fig. 1:**
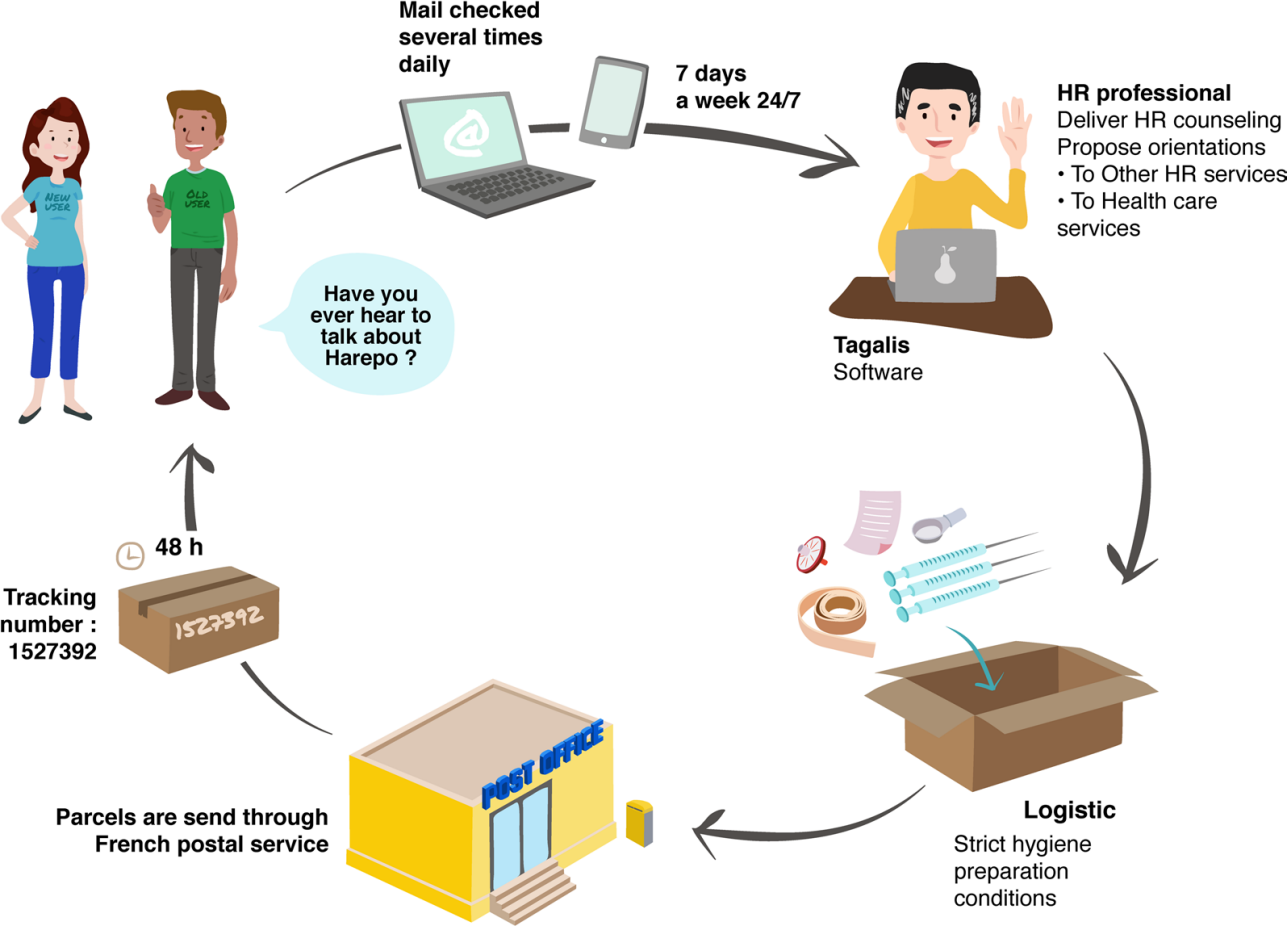
HaRePo Workflow

The remote harm reduction program, called HaRePo, was initiated in Paris (France), by the association SAFE in 2011, and is the first of its kind in the world [20]. It is free and intended for PWUD who have difficulty accessing HR tools and advice provided in specialized structures in France such as CAARUD (Centre d'Accueil et Accompagnement à la Réduction de Risques pour les Usagers de Drogues) and CSAPA (Centre de Soins, d'Accompagnement et de Prévention en Addictologie). The remote HR program does not require a physical meeting with the users. It is accessible by telephone and/or e-mail. An HR professional welcomes the users, provides advice and support and refers them to health care services and any other services adapted to their needs. Following the exchanges between the users and the professional, HR paraphernalia is registered and prepared under strict hygienic conditions. A package is personalized according to the person's needs and sent by the French Post Office to users in metropolitan France and overseas (Fig. 1). From 2011 to 2020, 2968 users have benefited from the program and 17,477 parcels have been sent, representing more than 2.6 million syringes and 10.2 million HR tools. The program has beneficial effects on users' practices and their perception of their health status. In an evaluation recently published, program users reported reduced risky practices when using drugs. Notably, the percentage of people who, after joining the program, never reuse and/or share HR tools increased significantly. In addition, some of them reported that their general physical condition (venous condition, injection sites, swelling of arms, legs and hands) had improved [20]. Remote HR is complementary to specialized structures and other HR programs and is part of a general HR policy for all users.

Founded in New York City, NEXT Distro “NEXT” became the first legal online and remote syringe service program in the USA. NEXT operates in locations where there is no pre-existing HR service provider by delivering supplies in discreet packaging through the United States Postal Service. The application for NEXT’s remote service is publically accessible on its website and staff geolocate each incoming applicant’s zip code with a directory of HR providers to ensure there is no geographic duplication. Once accepted into the program, NEXT uses encrypted phone, text, and email channels to securely communicate with program participants. Supplies can be ordered once per month through a web-based ordering system using a URL that is only disclosed to approved participants. NEXT also provides a complimentary naloxone-mailing program for individuals who are friends and family of PWUD and unable to access naloxone through traditional in-person methods. To advance remote HR services in the USA, NEXT has program partners in 30 states who use their centralized website to access remote HR services. To advance drug user health information using the internet, NEXT has consolidated safer injection, disease transmission prevention, sexual health, and treatment resources which are open access on their organization’s website resource page.

The aim of the study is therefore to test if and possibly how much the activity of two independent remote HR services located in France and in the USA (HaRePo and NEXT Distro [19, 20]) were impacted by the COVID-19 crisis and how they modified their performance to limit the effects of COVID-19 restriction measures on PWUD.

## Methods

In order to assess the restrictive measures impact due to COVID-19 on the two HR programs, we analyzed three indicators: the number of new PWUD joining the program, the number of parcels sent per month and the number of naloxone units distributed. The data are recorded by each program for its own logistic and management, they are not publicly available but available from the corresponding author. To estimate whether the COVID-19 pandemic significantly impacted these three indicators, we compared using *t*-test the year 2019 and the year 2020 for both structures. We considered the differences to be significant when *p*-values < 0.05. We used the year 2019 as reference period because the NEXT Distro program started recently and data of previous years were not available. All analysis were performer using RStudio 2022.07.1.

## Results

**Fig. 2:**
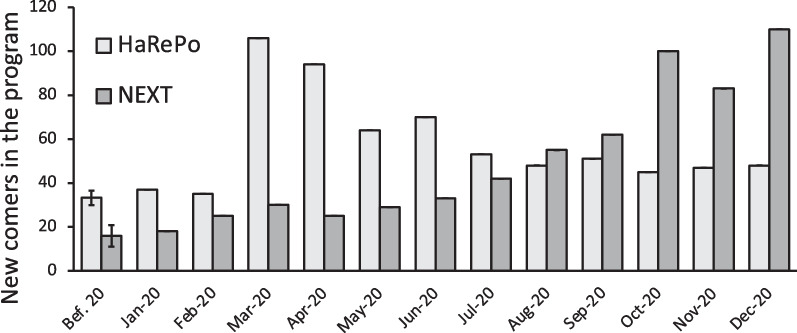
Numbers of newcomers in HaRePo and NEXT

In 2020, a significant increase of new PWUD entering the HaRePo or the NEXT Distro program compared to 2019 was observed with *p*-value < 0.005 for both structure (+ 77.6% for HaRePo and + 247.7% for NEXT Distro Table 1). We observed in Fig. 2 that in January and February 2020, for both programs, the number of new users joining per month was rather stable compared to previous years. In March, HaRePo faced a very large increase (300%) compared to the reference period. After March, the newcomers entering HaRePo decreased until July where they stabilized to 47.8 newcomers per month between August and December 2020. NEXT Distro also faced a significant increase, but the temporal dynamic was different. Indeed, in this case the increase started slightly in March but then increased every month until October when it reached 100 newcomers in the program which represents a 629% increase compared to the reference period. During the last month of the year the number of newcomers entering the NEXT Distro program stabilized around 100.

**Table 1:** Comparison of the new comers in the program, the parcel send per month, the parcel send per user enrolled and the naloxone distributed for the two structures (mean [CI 95%])

Years	HaRePo				NEXT Distro			
	New comers in the program per month	Parcels send per month	Parcels send per month by PWUD ordering	Naloxone distributed per parcel	New comers in the program per month	Parcels send per month	Parcels send per month by PWUD ordering	Naloxone distributed per parcel
2019	32.8 [25.0–40.5]	260.8 [250.9–270.8]	1.27 [1.25–1.29]	0.4 [0–1.1]	14.7 [12.3–17.0]	68.4 [58.1–78.8]	1.20 [1.15–1.26]	479.1 [301.9–656.3]
2020	58.2 [44.2–72.1]	372.3 [339.5–405.2]	1.41 [1.18–1.65]	9.4 [4.5–14.3]	51.0 [31.1–70.9]	213.0 [148.6–227.4]	1.14 [1.12–1.17]	667.2 [443.0–891.3]

**Fig. 3:**
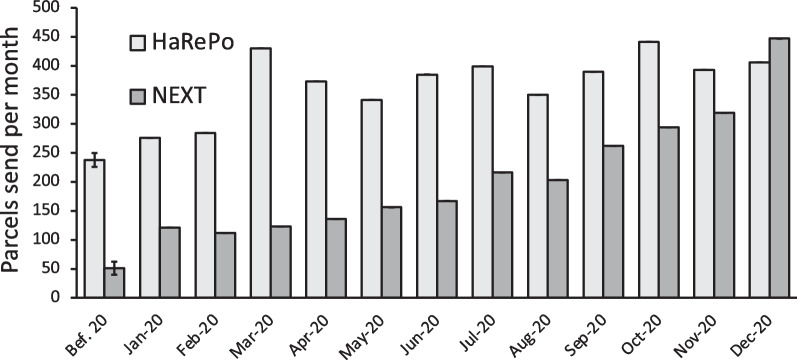
Number of parcels sent per year

The number of parcels sent per month (Fig. 3) is a good indicator of the entire activity of the programs because it represents the demand for all beneficiaries: the new comers and the demand of the PWUD already in the programs. As for the new comers, both structures faced a significant increase during the year 2020 compared to 2019 with *p*-value < 0.001 for both structures (+ 42.7% for Harepo and + 211.3% for NEXT Distro, Table 1). HaRePo faced a large and abrupt increase of activity during March after two months of relatively stable activity compared to the reference period. The HaRePo activity stays relatively stable after March but nearly doubled compared to the reference period. For NEXT Distro, the activity tends to increase since January 2020. Moreover, during the second half of 2020 the number of parcels sent per month increased again reaching on average 290.2 parcels sent which correspond to an increase of 567% compared to the reference period. When we calculated the numbers of parcels send per month and per PWUD enrolled in the program for the same month, we observed no significant difference between 2019 and 2020 for HaRePo (*p* = 0.21, Table 1) but for NEXT Distro a slight decrease is observed (1.20 for 2019 and 1.14 for 2020, *p* < 0.05, Table 1). This means that the increase of the number of parcels sent per month observed in both structures is only due to the increase of PWUD benefiting from the programs and not due to an increase of parcels sent per PWUD. Finally, it is interesting to note that the number of parcels sent per month and per PWUD was on the same order of magnitude for both structures (Table 1).

**Fig. 4:**
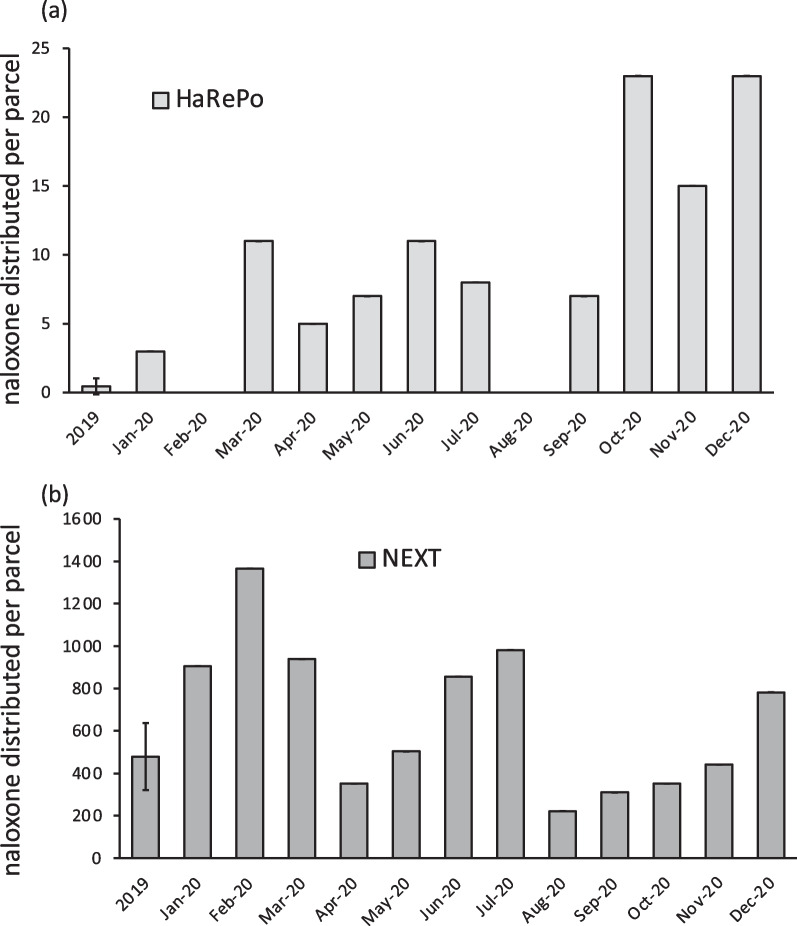
Number of naloxone distributed per parcel **a** HaRePo **b** NEXT

The naloxone distribution for HaRePo (Fig. 4a) was quite variable with a significant increase during the year 2020 (9.4 ± 4.3) compared to the year 2019 (0.45 ± 0.59) (*p*-value < 0.005). Despite this significant increase, the distribution of naloxone by HaRePo stayed relatively limited. Indeed, NEXT Distro distributed much more naloxone with 479.1 ± 157.8 naloxone doses sent per month during the year 2019 (Fig. 4b). The naloxone distribution for NEXT Distro increased during the three first months of 2020 with up to 1,365 doses distributed in February. After this period, it was variable with values ranging from 222 to 982 naloxone doses sent per month. Nevertheless, no significant differences between year 2019 and the year 2020 were detected by the *t*-test (*p*-value = 0.16).

## Discussion

In this study, we showed that the activity of two remote HR programs set in two different countries increased in 2020 compared to 2019, both because of significant newcomers joining in during the period but also because of an increase of their entire activity as showed by the number of parcels sent per month (Fig. 3). For HaRePo, the increase started drastically in March 2020 corresponding to the strict lockdown across the entire country of France, where the program is based. For NEXT Distro, the increase is less abrupt from one month to another but still significant. The difference between the two countries might be due to the different COVID-19 restrictions taken. Furthermore, because NEXT Distro was a newer organization when COVID-19 hit, there was less infrastructure to scale up and adequately respond to the emerging needs of the public. Indeed, in the USA the state and local governments are those who make decisions on lockdowns and how strict they should be whereas in France this decision is taken by the President and the government and was applied to the entire country. Therefore, the HR needs due to COVID-19 restriction were more abrupt in France than in the USA. As a consequence, some classical HR structures in France were restricted very early in 2020. Since NEXT Distro operates out of New York City, the lockdown impacted its activity in March 2020. Although HR services were deemed “essential” by the New York Department of Health, inadequate staffing and loss of volunteer support strained operations. In France, all the HR structures had to reduce their activities very abruptly in March 2020.

Another difference observed between the two structures is the naloxone distribution. HaRePo significantly increased its distribution during the year 2020 but the naloxone program started only in 2019, and therefore, the value considered for the reference period compared to 2020 is very limited. Moreover, in France, the availability of naloxone and the related publicity campaigns were carried out in 2019/2020 despite Naloxone is available in take-home formulation since 2016. Thus, information and communication for general public and training associations staff were more accessible since this date. In the USA the naloxone availability and publicity campaigns started in roughly two decades ago but with large differences between states. NEXT Distro has been distributing naloxone since it started its HR activities in 2018. This probably explains why the naloxone distribution by NEXT Distro is superior than with HaRePo but we cannot exclude that it might also be due to the very different epidemiology of opioid-related overdoses in these two contexts. The trends over 2020 are much more complex for NEXT Distro. There is an increasing trend of the naloxone distribution per month in 2020 compared to 2019 but the high month to month variability which results in a large confidence interval for the years 2019 and 2020 making this trend non-significant. Due to naloxone’s cost, NEXT Distro was forced to limit access which may explain why the trend is not significant as it became a function of supply and not demand.

Nevertheless, both structures were deeply impacted by the restrictions due to COVID-19 pandemic and increased their activities. This increase is likely a response to the reduced activities of other classical HR structures around the countries which had to reduce or at least adapt their activities. This suggests that remote HR policies like HaRePo and NEXT Distro were key programs to face the COVID-19 restrictions impact on HR policies both in France and in the USA. The fact that those two independent programs based on similar protocols (i.e., order online or by phone and paraphernalia send by mail) are identified as key responses during COVID-19 in two different countries with different HR policies suggest that remote approach is an valid option to face big events like the COVID-19 pandemic [17]. Even though tremendous efforts have been made by other classical HR structures, the remote approach is an effective alternative. For instance, ﻿social distancing measures may feel dehumanizing and limit the role of SSPs as safe spaces for participants [10]. Telehealth systems are useful but do not deliver paraphernalia [13]. Being 24/7, HR services are very flexible which seems an important positive aspect to reach PWUD and develop a successful HR policy [21].

HaRePo and NEXT Distro can therefore be considered as remote HR service and is part of a broader category called home-delivery services [22–24]. Home-delivery services are generally designed to help difficult-to-reach populations to access HR services like women [25] or populations living in rural areas [26]. A larger study on how COVID-19 related restrictions quantitatively impacted the activities of home-delivery services would be of interest to broaden our conclusions and show that those types of approaches must be developed in other countries because (i) they compliment classical HR services and (ii) they are key structures to face big-event crisis that will likely occur in the future. Nevertheless, it is important to note that remote HR structures cannot be only considered as take-home medications [27, 28]. Indeed, first there is no need of medical prescription to access paraphernalia whereas it can be the case for some take-home medications. Moreover, PWUD within a remote HR service do not need to travel to get their paraphernalia.

Nevertheless, remote HR services such as HaRePo and NEXT Distro can face limitations. The very first one is that the users need a delivery address, which could be difficult for homeless populations. Nevertheless, some options are available. In France, HaRePo benefits from general policies for homeless people: a special structure called CCAS (Communal Center for Social Action; Centre Communal d’Action Social in French) provide an address to homeless people and can receive letters or parcels and keep them until the person comes to collect it. Moreover, delivery is possible at a third party in France that could be a friend, family member or even a shop. In the USA, some HR structures exist and can be used for HR paraphernalia [29]. Moreover, packages can be delivered to homeless and unstably-house people via the USPS (United States Postal Service) through a service called “General Delivery.” In France it is also possible to mail the parcel to the post office closest to users. In France and USA users can pick parcels up with a piece of identification.

Another possible improvement for remote HR services is to also provide telehealth. Indeed, a previous study suggested that multi-services are more efficient than single services to help PWUD [30]. Finally, as with most HR services, remote HR structures may be threatened by the risk of funding limitations [31]. HaRePo already partially faced this situation since it has received orders from other EU countries but does not have funding to send parcels outside of France.

It is important to note that our study has some limitations. In particular the comparisons between the two structures because COVID-19-related restrictions were different in both countries. Moreover, both structures are based on similar approaches but with their own specificities, e.g., local cultures and politics, profiles of PWUD, and age of the services. Thus, even though remote HR services were an efficient response to provide HR equipment to PWUD during COVID-19-related movement restrictions in France and in the USA, an implementation of a similar service in another country has to take into account the local specificities to be fully efficient.

## Conclusions

To conclude, this study provides evidence of an increased activity of two independent remote HR services in two different countries with different responses to the COVID-19 crisis. Our results suggest that remote approach buffered the effect of reduced activities of other classical HR services on PWUD. We therefore consider that these approaches are not only key structures to help difficult-to-reach populations to access HR services but also to face big events with impacts on HR service organization.

## Data Availability

The datasets generated during and/or analyzed during the current study are available from the corresponding author on reasonable request.

## Abbreviations

HaRePo: Harm reduction by post
HR: Harm reduction
PWUD: People who use drugs
CAARUD: Centre d'Accueil et d'Accompagnement à la Réduction des Risques des Usagers de Drogues
CCAS: Centre Communal d’Action Sociale
CSAPA: Centres de Soins, d'Accompagnement et de Prévention en Addictologie
SSPs: Syringe Service Programs
USPS: United States Postal Service
